# Hesperetin—Between the Ability to Diminish Mono- and Polymicrobial Biofilms and Toxicity

**DOI:** 10.3390/molecules27206806

**Published:** 2022-10-11

**Authors:** Tamara Carević, Marina Kostić, Biljana Nikolić, Dejan Stojković, Marina Soković, Marija Ivanov

**Affiliations:** 1 Department of Plant Physiology, Institute for Biological Research “Siniša Stanković”, National Institute of Republic of Serbia, University of Belgrade, Bulevar Despota Stefana 142, 11000 Belgrade, Serbia; 2 Department of Microbiology, Faculty of Biology, University of Belgrade, Student Square 16, 11000 Belgrade, Serbia

**Keywords:** hesperetin, flavonoid, *Candida albicans*, antifungal, toxicity, antibiofilm, polymicrobial biofilm

## Abstract

Hesperetin is the aglycone of citrus flavonoid hesperidin. Due to the limited information regarding hesperetin antimicrobial potential and emerging need for novel antimicrobials, we have studied its antimicrobial activity (microdilution assay), antibiofilm activity with different assays in two models (mono- and polymicrobial biofilm), and toxicity (MTT and brine shrimp lethality assays). Hesperetin inhibited growth of all *Candida* isolates (minimal inhibitory concentration, MIC, 0.165 mg/mL), while it’s inhibitory potential towards *Staphylococcus aureus* was lower (MIC 4 mg/mL). Hesperetin (0.165 mg/mL) reduced ability of *Candida* to form biofilms and moderately reduced exopolysaccharide levels in biofilm matrix. Effect on the eradication of 24 h old *C. albicans* biofilms was promising at 1.320 mg/mL. Inhibition of staphylococcal biofilm formation required higher concentrations of hesperetin (<50% inhibition with MIC 4 mg/mL). Establishment of polymicrobial *C. albicans*-*S. aureus* biofilm was significantly inhibited with the lowest examined hesperetin concentration (1 mg/mL) in crystal violet and CFU assays. Hesperetin toxicity was examined towards MRC-5 fibroblasts (IC_50_ 0.340 mg/mL) and in brine shrimp lethality assay (LC_50_ > 1 mg/mL). Hesperetin is efficient in combating growth and biofilm formation of *Candida* species. However, its antibacterial application should be further examined due to the cytotoxic effects provoked in the antibacterial concentrations.

## 1. Introduction

Flavonoids are a large group of phenolic constituents found in plants. They are divided into several subgroups, of which flavonols are the most extensively studied. On the other hand, the subgroup of flavanones, to whom hesperetin belongs, has attracted less scientific attention related to human health and accordingly the health effects of flavanones are still largely unknown. Flavanones occur almost exclusively in citrus fruits. The highest concentrations are found in the solid tissues, but concentrations of several hundred milligrams per liter are present in the juice as well [1]. Most human beings are exposed to flavonoids daily, and therefore, their impact on human health is of relevance for scientific community and pharmaceutical industry.

It has been shown previously that hesperetin inhibits chemically induced mammary, urinary bladder, and colon carcinogenesis in experimental animals [2]. Hesperetin also possess some antioxidant activity, although this activity is weaker compared with many other polyphenols [3]. New findings showed that the antioxidant activity of hesperetin was not only limited to its radical scavenging activity, but it augmented the cellular antioxidant defense via the ERK/Nrf2 signaling pathway as well [4]. Other possible effect of hesperetin is on lipid metabolism; it regulates apolipoprotein B secretion by HepG2 cells, possibly through inhibition of cholesterol ester synthesis [5]. Hesperetin showed good health beneficial features due to its role in combating diabetes and its complications [6].

*Candida* species are one of the most common fungal threats to the human health worldwide. In the US, candidemia, *Candida* spp. infection in the blood, is in the top four most common nosocomial bloodstream infections in the country [7]. The most common cause of candidemia is *Candida albicans* followed by *C. tropicalis*, *C. parapsilosis*, and *C. glabrata*, as recorded among intrahospital patients in China [8].

Due to the increasing incidence of the fungal infections and limits of the current antifungal palette we are currently witnessing the increasing worldwide need for the development of novel antifungals. Different fungal traits are being investigated as potential antifungal targets including fungal ability to form biofilms [9], the trait that is being linked to the higher mortality rates in patients with candidemia [10].

Causative agents of persistent chronic infections worldwide are as well bacteria organized into bacterial aggregates or biofilms, which are highly resistant to available antibiotics. Polymicrobial biofilm community is defined as a group of various microorganisms (bacteria, virus, and fungi) present on different surfaces and coated within a hydrated matrix, often composed of polysaccharides and produced by its microbial constituents [11]. The presence of polymicrobial infections has important implications in disease management because it can modify the clinical course of the disease [12]. A disease related to polymicrobial infections from several infective agents is referred to as complex, complicated, mixed, multiple, synergistic, and concurrent clinical or pathological manifestation. This impacts the choice of antimicrobial therapy and the response to be anticipated, especially since the belonging pathogens are usually resistant to antimicrobial agents [13,14]. Therefore, use of natural agents that prevent the formation of biofilms is an extremely important strategy in the fight against the causative agents of both mono and polymicrobial biofilm associated infections.

Bearing in mind that hesperetin has not been extensively studied yet, the aim of the current study was to estimate the antifungal and antibacterial, actually anticandidal and antistaphylococcal properties of this compound, to investigate its antibiofilm properties, including the effect on polymicrobial biofilm, and to evaluate its toxicity in vitro on selected cell line, and in vivo on brine shrimps.

## 2. Results

### 2.1. Antimicrobial Activity

Hesperetin has exhibited promising anticandidal potential as determined in the microdilution assay. Minimal inhibitory concentration (MIC) and minimal fungicidal concentration (MFC) values determined were 0.165 mg/mL and 0.330 mg/mL, respectively, towards both *albicans* and non-*albicans Candida* strains examined in the study (Table 1).

In the case of antibacterial activity, hesperetin showed lower activity compared to antifungal, with MIC value 4 mg/mL and minimal bactericidal concentration (MBC) 8 mg/mL for *Staphylococcus aureus* (Table 2).

### 2.2. Antibiofilm Spectrum of Hesperetin

#### 2.2.1. Hesperetin Inhibited *Candida Albicans* Biofilm Forming Ability

**Figure 1:**
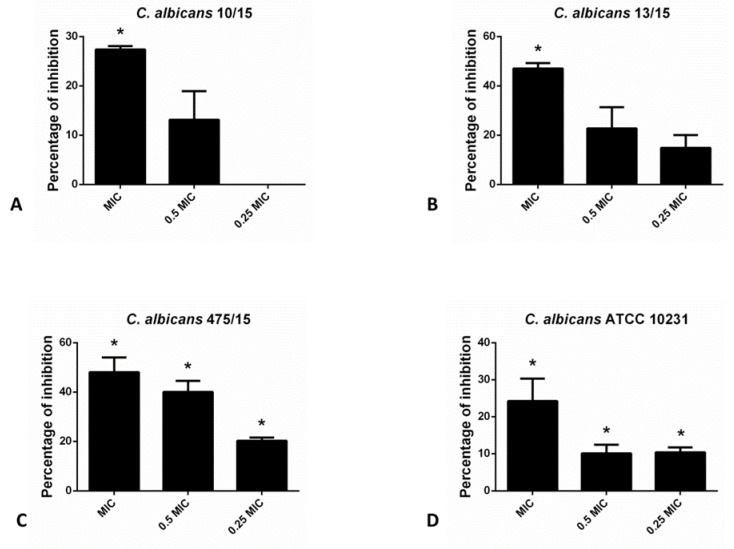
Inhibition of biofilm formation in different *C. albicans* strains: (**A**)- *C. albicans* 10/15; (**B**)- *C. albicans* 13/15; (**C**)- *C. albicans* 475/15 and (**D**)- *C. albicans* ATCC 10231 after application of hesperetin in concentrations 0.25 MIC (0.041 mg/mL), 0.5 MIC (0.082 mg/mL) and MIC (0.165 mg/mL). Mean values of triplicate independent experiments ± SD are shown. * *p* < 0.05 compared to control.

As indicated in the Figure 1, hesperetin has exhibited significant ability to reduce biofilm forming potential of different *C. albicans* species, as determined in the crystal violet (CV) assay. Incubation of these yeasts with MIC of hesperetin has reduced biofilm formation for more than 40%, as in the case of *C. albicans* 13/15 and *C. albicans* 475/15. More than 20% biofilm inhibition was noticed for other *C. albicans* strains examined, upon co-incubation with MIC of hesperetin.

#### 2.2.2. Hesperetin Interfered with Non-Albicans Candida Biofilm Forming Ability

**Figure 2:**
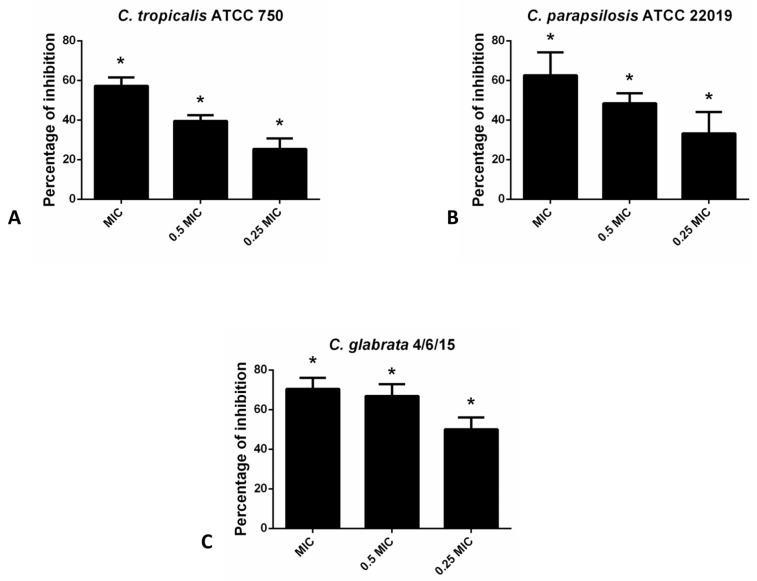
Inhibition of biofilm formation in different non-*albicans Candida* strains: (**A**)- *C. tropicalis* ATCC 750; (**B**)- *C. parapsilosis* ATCC 22019; (**C**)- *C. glabrata* 4/6/15 after application of hesperetin in concentrations 0.25 MIC (0.041 mg/mL), 0.5 MIC (0.082 mg/mL) and MIC (0.165 mg/mL). Mean values of triplicate independent experiments ± SD are shown, * *p* < 0.05 compared to control.

In the case of non-*albicans Candida* species in the CV assay, hesperetin has exhibited the most profound inhibitory effect on the biofilm forming capacity of *C. glabrata* 4/6/15 (70.4% and 66.9% inhibition with MIC and 0.5 MIC of hesperetin, respectively). Likewise, biofilm formation of other non-*albicans* species such as *C. parapsilosis* ATCC 22019 and *C. tropicalis* ATCC 750 was interrupted significantly with the presence of hesperetin (Figure 2).

#### 2.2.3. Hesperetin Efficiently Eradicated 24 h Old *C. albicans* Biofilms

**Figure 3:**
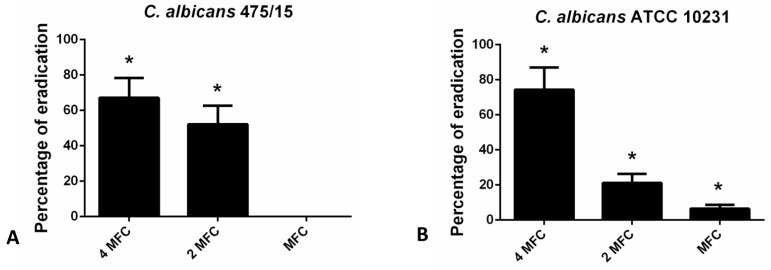
Eradication of 24 h old (**A**)- *C. albicans* 475/15; (**B**)- *C. albicans* ATCC 10231 biofilms after application of hesperetin in concentrations MFC (0.330 mg/mL), 2 MFC (0.660 mg/mL) and 4 MFC (1.320 mg/mL). Mean values of triplicate independent experiments ± SD are shown, * *p* < 0.05 compared to control.

For the eradication of fungal biofilms higher concentration (4 MFC, 2 MFC and MFC) were examined in CV assay (Figure 3). The highest applied concentration (4 MFC) exhibited the strongest biofilm eradicating potential (24 h old biofilms were eradicated for more than 60% in the case of both strains tested).

#### 2.2.4. Could the Hesperetin Antibiofilm Activity Be Attributed to Reduction of Exopolysaccharide Matrix?

**Figure 4:**
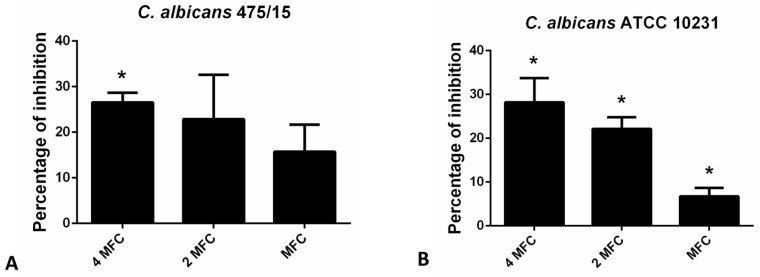
Inhibition of biofilm matrix exopolysaccharides as determined in the Congo red binding assay for (**A**)- *C. albicans* 475/15; (**B**)- *C. albicans* ATCC 10231 after application of hesperetin in concentrations MFC (0.330 mg/mL), 2 MFC (0.660 mg/mL) and 4 MFC (1.320 mg/mL). Mean values of triplicate independent experiments ± SD are shown, * *p* < 0.05 compared to control.

Congo red binding assay was used to estimate the levels of exopolysaccharides (EPS) in fungal biofilm matrix that remain upon application of hesperetin (Figure 4). Reduction of EPS in the matrix did not exceed 30% in the presence of hesperetin, unlike reduction of total biofilm biomass determined in the CV assay (Figure 3), suggesting that the EPS reduction potential is not the only antibiofilm mechanism, but other ones are also involved in the hesperetin biofilm eradicating capability.

#### 2.2.5. Hesperetin Inhibited *Staphylococcus Aureus* Biofilm Forming Ability

**Figure 5:**
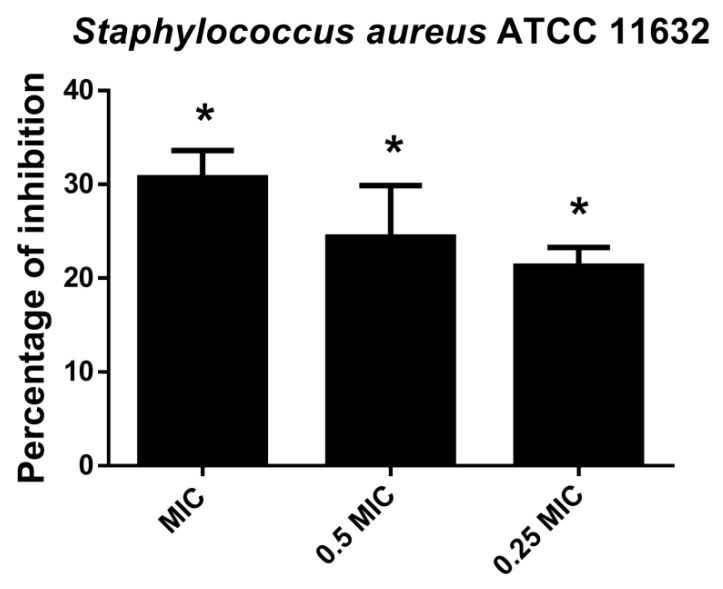
Inhibition of biofilm formation in *S. aureus* ATCC 11632 after application of hesperetin in concentrations 0.25 MIC (1 mg/mL), 0.5 MIC (2 mg/mL) and MIC (4 mg/mL). Mean values of triplicate independent experiments ± SD are shown. * *p* < 0.05 compared to control.

CV assay indicated that hesperetin affected *S. aureus* attachment ability and consequently inhibited biofilm formation in a dose-dependent manner (Figure 5). Inhibition of biofilm formation at MIC value was 30.7%, while 21.3% of biofilm inhibition could be achieved with hesperetin in 0.25 MIC (1 mg/mL).

#### 2.2.6. Hesperetin Impaired Polymicrobial *C. albicans*-*S. aureus* Biofilms

**Figure 6:**
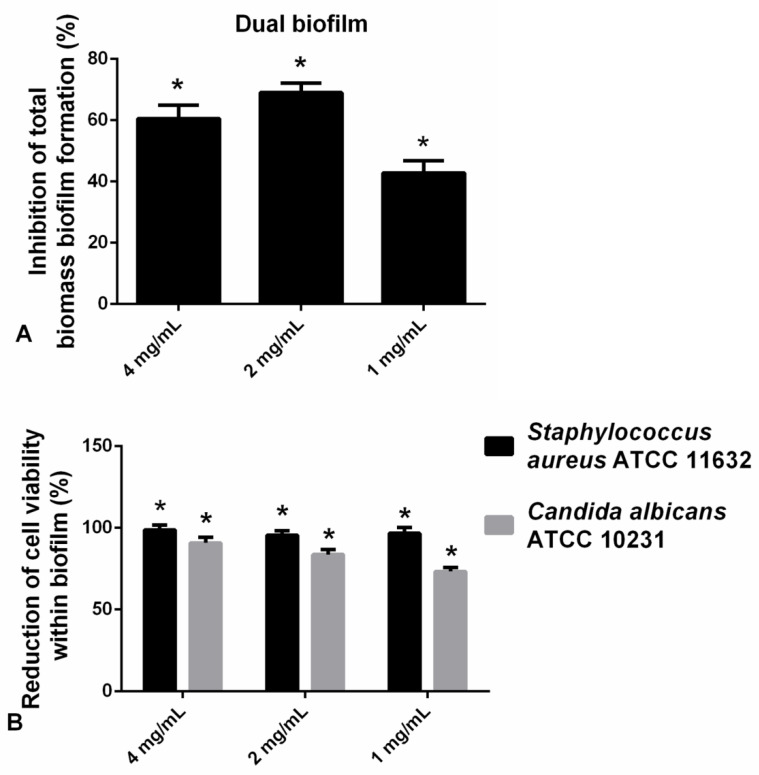
(**A**) Inhibition of polymicrobial *C. albicans*-*S. aureus* biofilm formation after application of hesperetin in concentrations 4 mg/mL, 2 mg/mL, and 1 mg/mL—CV assay. (**B**) Reduction of cell viability of *S. aureus* ATCC 11632 and *C. albicans* ATCC 10231 in polymicrobial biofilm after treatment with hesperetin (4 mg/mL, 2 mg/mL, and 1 mg/mL)—CFU assay. Mean values of triplicate independent experiments ± SD are shown, * *p* < 0.05 compared to control.

The application of hesperetin (2 mg/mL) against polymicrobial biofilm consisted of *C. albicans* and *S. aureus* has significantly reduced biofilm biomass as determined in the CV assay (Figure 6A). Additionally, in the case of the *S. aureus*-*C. albicans* mixed biofilm, the viable cells of both microorganisms were found to be reduced for more than 90% at all tested concentrations, as determined in the CFU assay (Figure 6B). Significant reduction in the viable cells of *S. aureus* and *C. albicans* was observed when compared to the control (Figure 6B).

### 2.3. Toxicity of Hesperetin

Risk assessment of potential hesperetin use was provided by in vitro cytotoxicity test (MTT) and in vivo brine shrimp lethality assay. Cytotoxicity of hesperetin was noticed towards MRC-5 fetal lung fibroblasts with IC_50_ 340 µg/mL. On the other hand, its LC_50_ in the brine shrimp lethality assay was not determined in the applied concentration range, but was >1000 µg/mL (Table 3).

## 3. Discussion

In this study hesperetin has exhibited promising anticandidal activity, displayed towards both *Candida albicans* and non-*albicans Candida* strains. Previous study has examined effect of hesperetin towards *C. glabrata* and found it inefficient in concentrations up to 0.083 mg/mL [15], which is lower than inhibitory concentration established in this study (0.165 mg/mL). Hesperetin antifungal potential is still much lower compared to the antifungal activity of ketoconazole, positive control used in the antimicrobial assay. However, there are different side effects and limitations linked with the usage of current antifungal therapeutics. For example, ketoconazole can cause liver dysfunction, skin or scalp irritation or burning, and local allergic reaction (contact dermatitis). Bearing in mind side effects and limitations of current antifungal pallet and an emerging demand for the development of novel antifungal therapeutics [9], anticandidal potential of hesperetin should be further explored, and has the potential to be utilized for the design of novel antifungal strategies.

According to the author’s best knowledge, this is the first study elucidating wide inhibitory potential of hesperetin towards *Candida* biofilms formed by seven different *Candida* strains in total. This molecule was active against biofilm formation of both *C. albicans* and non-*albicans Candida* strains. Additionally, hesperetin was not efficient in prevention of biofilm establishment only, but also in its eradication. However, for biofilm eradication hesperetin was applied in concentrations higher than 0.330 mg/mL, which almost reaches up to its cytotoxic concentration (IC_50_ 0.340 mg/mL towards MRC-5). The antibiofilm properties of hesperetin could be utilized for the purpose of preventing the formation of biofilms rather than eradicating them, since this flavonoid can efficiently prevent biofilm formation in concentrations that are much lower than the ones needed for biofilm eradication. The antibiofilm mechanism can be attributed to the reduction of total biofilm biomass and exopolysaccharide production in biofilm matrix. Natural products have been studied for decades now as biofilm inhibitors [16], with the search continuing up to today. Hesperetin antibiofilm potential highlighted in this study is one more reason to further examine this molecule as a promising anticandidal agent. 

On the other hand, antistaphylococcal potential of hesperetin determined in this study was low. Previous study of hesperetin has indicated its low inhibitory potential towards *S. aureus* (MIC > 1 mg/mL) [17], as confirmed also in this study (MIC > 4 mg/mL). Moreover, stronger antibacterial potential for hesperetin was noticed in the study of Choi et al. [18], who determined MIC at 0.125 mg/mL, and in the study of Ivanov et al. [19], with MIC 0.5 mg/mL, both towards *S. aureus*. In this previous study [19] hesperetin exhibited identical antibacterial potential as its glycoside hesperidin, suggesting that sugar moiety does not have impact on antibacterial properties of this flavonoid. As indicated in the literature, hesperetin can also inhibit bacterial biofilms. Previous study [19] has confirmed significant antibiofilm potential of hesperetin towards *P. aeruginosa* IBRS P001, with hesperetin being among the strongest antibiofilm polyphenols among the 11 compounds examined. Another study has established minimal biofilm inhibitory concentrations of hesperetin (MBIC_50_) towards *S. aureus* RN4220 and *S. aureus* SA1199B at 4 µg/mL and 32 µg/mL, respectively [20]. However, hesperetin in this study was able to inhibit *S. aureus* biofilm by applying higher concentrations (1–4 mg/mL), suggesting less promising potential for this flavonoid to combat bacterial biofilms, compared to the ones formed by pathogenic fungi.

According to the authors’ best knowledge, this is the first study of hesperetin effect towards polymicrobial biofilms. This flavonoid has exhibited great potential in reducing numbers of viable cells of both *C. albicans* and *S. aureus* in mixed biofilm community, as well as in the reduction of total biofilm biomass. Nowadays, we are struggling with the urging need for the discovery of agents efficient towards polymicrobial biofilms since majority of antibiofilm studies have been focused on monobiofilm models, despite the fact that the natural biofilms are often polymicrobial [21]. However, inhibitory activity displayed by hesperetin against *C. albicans-S. aureus* biofilm is of high importance for the development of novel antimicrobials that could combat the dual species biofilms. Its inhibitory effect on polymicrobial biofilms is linked to reduction in both total biofilm biomass and in cell viability.

The evaluation of cytotoxicity for potential pharmaceutic agent is an essential step in biomedical research and represents a primary consideration during the drug selection. Likewise, the first step in the development of novel antimicrobial drugs includes in culture toxicity studies on human cells [22]. Accordingly, we have examined cytotoxic effects of hesperetin on normal fibroblasts (MRC-5 cells) and found its IC_50_ 0.340 mg/mL, suggesting that application of hesperetin as antifungal agent might be safe, since MIC towards *Candida* species were below the toxic levels (0.165 mg/mL). However, antibacterial application of hesperetin requires concentrations that are few magnitudes higher than the cytotoxic, implying that hesperetin could not be considered as a safe antibacterial agent, at least as determined in experimental models used in this study. On the contrary, in vivo toxicity test on brine shrimps has not determined any toxic effect in the applied concentration range (LC_50_ > 1 mg/mL), but further study is needed, since determined MIC for *S. aureus* is four times higher than the threshold of tested toxicity.

Hesperetin is an understudied flavonoid that deserves much more attention due to its wide antifungal potential focused towards both planktonic and biofilm fungal cells. Moreover, its inhibitory effect on the polymicrobial biofilms is of great interest for the further studies and development of novel antimicrobial strategies. On the other hand, its activity towards *S. aureus* is negligible. Toxicity studies of hesperetin indicate that this molecule could be considered safe (up to threshold of toxicity screened in brine shrimp lethality assay) or safe up to certain concentrations (MRC-5 cytotoxicity). With the emerging lack of efficacy of current antimicrobial pallet, compounds such as hesperetin gain a lot of attraction and could direct the antimicrobial research towards flavonoids, as a promising group of natural bioactive molecules. However, toxicity studies should be done in more details in order to completely elucidate risk assessments of hesperetin application. Moreover, further studies are needed in order to provide more detailed insight into mechanisms of hesperetin antimicrobial activity.

## 4. Material and Methods

### 4.1. Anticandidal Assay

Microdilution assay [23] with some modification was used. Strains used in the assay were clinical isolates: *C. albicans* 10/15, *C. albicans* 13/15, *C. albicans* 475/15, *C. krusei* H1/16, and *C. glabrata* 4/6/15 and reference strains: *C. albicans* ATCC 10231, *C. parapsilosis* ATCC 22019, and *C. tropicalis* ATCC 750.

MIC and MFC were determined by microdilution assay. First, yeast cultures were adjusted to McFarland 0.5 with sterile PBS. The 96-well microtiter plates with serially diluted tested agents in liquid broth were incubated at 37 °C for 24 h. Upon incubation, MICs and MFCs were determined. The lowest concentrations without microscopically observed growth were considered as MIC. For microscopic determination of growth, inverted microscope Nikon Eclipse TS2 (Amsterdam, The Netherland) was utilized and fungal growth in the wells of 96-well microtiter plates compared to the control (untreated yeast cells) was examined. MFC values were observed as concentrations without visible growth after serial sub-cultivation of 10 µL of samples at 37 °C for 24 h. Ketoconazole (SigmaAldrich, Darmstadt, Germany) was used as positive control.

### 4.2. Anti-Staphylococcal Activity

Strain used in the assay was reference strain *S. aureus* ATCC 11632. MIC and MBC were calculated as described previously [23].

### 4.3. Crystal Violet Antibiofilm Assay

As for the inhibition assay, *Candida* strains or *S. aureus* were incubated with MIC and sub-MIC of the tested compounds in YPD and TSB, respectively, at 37 °C for 24 h [24]. Upon incubation, 96-well microtiter plates were washed twice with sterile PBS and fixed with methanol during 10 min. After methanol removal the plate was air-dried. The biofilm was stained with 0.1% crystal violet (SigmaAldrich, Germany) for 30 min. The plates were washed with water, air dried, and the stain was dissolved in 96% ethanol (Zorka, Sabac, Serbia). The absorbance was read at 620 nm on a Multiskan™ FC microplate photometer (Thermo Scientific™, Waltham, MA, USA).

As for the biofilm eradication assay *Candida* was incubated in YPD at 37 °C for 24 h. Upon incubation wells were washed with PBS and the remaining biofilm was treated with hesperetin (MFC, 2 MFC and 4 MFC) at 37 °C during another 24 h. Upon treatment, biofilm washing and staining was performed as described in the previous paragraph.

### 4.4. Congo Red Binding Assay

The estimation of hesperetin influence on biofilm EPS production was determined with the Congo red binding assay [24,25]. Biofilms were formed (37 °C, 24 h) and upon removal of planktonic cells they were incubated in the presence of hesperetin in concentrations MFC (0.330 mg/mL), 2 MFC (0.660 mg/mL) and 4 MFC (1.320 mg/mL) at 37 °C for another 24 h. Wells were washed with PBS and stained with 1% (*w*/*v*) Congo red (SigmaAldrich, Germany) in the dark for 30 min. Wells were aspirated and subsequently the bound dye was solubilized with 200 μL DMSO. Absorbance at 490 nm was measured in a microtiter plate reader Multiskan™ FC microplate photometer (Thermo Scientific™, Waltham, MA, USA) and the inhibition percentage of EPS production was calculated according to the following equation:

%Inhibition = [(A490_control_ − A490_sample_)/A490_control_] × 100 (1)

With A490_control_ representing the absorbance of the untreated biofilm and A490_sample_ the absorbance of the hesperetin treated sample. 

### 4.5. Polymicrobial Biofilm Inhibition—CV Assay

Dual biofilms [26] were formed in 96-well microtiter plates with adhesive bottom (Spektar, Čačak, Serbia) by adding 10 µL of each of *C. albicans* ATCC 10231 and *S. aureus* ATCC 11632, McFarland 0.5. Microorganisms were incubated in TSB and YPD medium at 37 °C for 24 h with 1 mg/mL, 2 mg/mL, and 4 mg/mL of hesperetin. The inhibition of biomass formation of dual biofilm was quantified by the crystal violet (CV) assay [23]. The CV absorbance was then measured spectrophotometrically at a wavelength of 620 nm using a microtiter plate reader (Multiskan FC Microplate Photometer, Thermo Scientific) and calculated as previously described [24].

### 4.6. Dual Biofilm Inhibition—CFU Assay

Dual biofilms were prepared as described above, on coverslips, and treated as in Harriott et al. [27], with some modifications. Biofilms (24 h old) pre-incubated with hesperetin were washed in 1XPBS to remove non-adherent cells. Cells were re-suspended in 1XPBS by sonicating for 10 min and followed by pipetting up and down. Dilutions of each sample were made and plated on HiCrome™ Candida Differential Agar (Himedia, Mumbai, India) and TSA (Torlak, Belgrade, Serbia) supplemented with Amphotericin B (0.025 mg/mL). The plates were incubated at 37 °C for 24 h. Bacterial and fungal colonies were counted the next day and the percentage of inhibition in the polymicrobial biofilm constituents under the influence of hesperetin was calculated.

### 4.7. Cytotoxicity

The cytotoxic effect of hesperetin was determined by MTT assay [28]. MRC-5 cells, human fetal lung fibroblasts, were inoculated into 96-well plates at a density 5 × 10^4^ cells/well and incubated 24 h to form a monolayer. After washing with PBS, the fresh medium containing different concentrations of hesperetin was added, and the incubation continued for 24 h. The MTT (final concentration 0.5 mg/mL) was then added, and the plates were incubated for additional 3 h. After incubation the medium was carefully removed and the formazan crystals were dissolved in DMSO. Cell viability was determined by measuring absorbance at 570 nm, using a Micro-plate reading Spectrophotometer (Thermo, Scientific) and IC_50_ value was calculated.

### 4.8. Brine Shrimp Lethality Assay

Brine shrimp lethality assay was conducted as previously described [29], with some modifications. Approximately 24 h after hatching, the phototropic nauplii were collected with a pipette from the lighted side and concentrated in a small vial. Then, ten brine shrimp were transferred to each well using adequate pipette. Then, every group of 10 Artemia aged 24 h was exposed to various concentrations of the hesperetin. The toxicity was determined after 24 h of exposure. The positive control was potassium dichromate K_2_Cr_2_O_7_ (Sigma Aldrich). The numbers of survivors were counted and percentage of deaths was calculated. Larvae were considered dead if they did not exhibit any internal or external movement during several seconds of observation. The final results are presented as LC_50_.

### 4.9. Statistical Analysis

All the experiments were performed in three repeats. All the data were calculated as a mean  ±  standard error and statistically analyzed using GraphPad PRISM 6 software. * *p* < 0.05 compared to control is considered significant. Student’s t-test was used.

## Figures and Tables

**Table 1:** Antifungal activity of hesperetin, results are in mg/mL. MIC- minimal inhibitory concentration; MFC- minimal fungicidal concentration. Different letters (a, b) in each row indicate a significant statistical difference between the samples (*p* < 0.05). MIC and MFC values are compared separately for each of the fungal strain tested.

Strain	Hesperetin		Ketoconazole	
	MIC	MFC	MIC	MFC
*C. albicans* 10/15	0.165 ^b^	0.330 ^b^	0.003 ^a^	0.050 ^a^
*C. albicans* 13/15	0.165 ^b^	0.330 ^b^	0.002 ^a^	0.050 ^a^
*C. albicans* 475/15	0.165 ^b^	0.330 ^b^	0.003 ^a^	0.006 ^a^
*C. albicans* ATCC 10231	0.165 ^b^	0.330 ^b^	0.002 ^a^	0.006 ^a^
*C. parapsilosis* ATCC 22019	0.165 ^b^	0.330 ^b^	0.003 ^a^	0.006 ^a^
*C. tropicalis* ATCC 750	0.165 ^b^	0.330 ^b^	0.002 ^a^	0.006 ^a^
*C. krusei* H1/16	0.165 ^b^	0.330 ^b^	0.002 ^a^	0.003 ^a^
*C. glabrata* 4/6/15	0.165 ^b^	0.330 ^b^	0.002 ^a^	0.003 ^a^

**Table 2:** Antibacterial activity of hesperetin, results are in mg/mL. MIC- minimal inhibitory concentration; MBC- minimal bactericidal concentration. Different letters (a, b) in each row indicate a significant statistical difference between the samples (*p* < 0.05). MIC and MBC values are compared separately.

Strain	Hesperetin		Streptomycin	
	MIC	MBC	MIC	MBC
*S. aureus* ATCC 11632	4.00 ^b^	8.00 ^b^	0.04 ^a^	0.10 ^a^

**Table 3:** Cytotoxicity/toxicity of hesperetin observed towards MRC-5 cells in vitro and brine shrimps in vivo (µg/mL).

Compound	MRC-5 (IC_50_)	Brine shrimp (LC_50_)
Hesperetin	340 ± 29 µg/mL	>1000 µg/mL
K_2_Cr_2_O_7_	*-*	<10 µg/mL

## Data Availability

Data is contained within the article.
